# A synergistic therapy against influenza virus A/H1N1/PR8 by a HA1 specific neutralizing single-domain V_L_ and an RNA hydrolyzing scFv

**DOI:** 10.3389/fmicb.2024.1355599

**Published:** 2024-04-19

**Authors:** Phuong Thi Hoang, Quynh Xuan Thi Luong, Ramadhani Qurrota Ayun, Yongjun Lee, Kwang-Ji Oh, Taehyun Kim, Taek-Kyun Lee, Sukchan Lee

**Affiliations:** ^1^Department of Integrative Biotechnology, Sungkyunkwan University, Suwon, Republic of Korea; ^2^Novelgen Co., Ltd., R&D Center, Suwon-si, Gyeonggi-do, Republic of Korea; ^3^Risk Assessment Research Center, Korea Institute of Ocean Science and Technology, Geoje, Republic of Korea

**Keywords:** bio-panning, synergistic effect, influenza virus, 3D8 scFv, neutralizing antibodies

## Abstract

The emergence of anti-influenza drug-resistant strains poses a challenge for influenza therapy due to mutations in the virus’s surface protein. Recently, there has been increasing interest in combination therapy consisting of two or more drugs as a potential alternative approach, aiming to enhance therapeutic efficacy. In this study, we investigated a novel synergistic therapy with a vertical effect using a single-domain VL-HA1-specific antibody against H1N1/PR8 and a horizontal effect using an RNA catalytic antibody with broad-spectrum influenza antiviral drug. We isolated a single-domain VL-HA1-specific (NVLH8) antibody binding to the virus particles showing a neutralizing activity against influenza virus A, specifically H1N1/PR8, as determined by the reduction in plaque number and lower viral HA protein expression *in vitro*. The neutralizing antibody likely prevented the viral entry, specifically at the viral genome-releasing step. Additionally, the 3D8 scFv hydrolyzed viral RNAs in the cytoplasm, including mRNA, vRNA, and cRNA in MDCK cells. The combined treatment of neutralizing antibodies for a vertical effect and 3D8 scFv for a horizontal effect produced a synergistic effect providing a novel approach against viral diseases when compared with a single treatment. Our results indicated that combining treatment, in particular two proteins exhibiting different mechanisms of action increased the antiviral activity against the influenza virus.

## 1 Introduction

Annually, seasonal influenza virus infections cause approximately 290,000–650,000 deaths worldwide (Iuliano et al., 2018). A yearly vaccination is required because of the viral HA and NA protein shift and drift. Influenza A is a segmented negative-stranded RNA virus whose replication cycle occurs in the nucleus (McGeoch et al., 1976). Each of the eight viral RNA gene segments is presented in a viral ribonucleoprotein complex (vRNP) composed of a viral RNA (vRNA) covered with nucleoprotein (NP) and heterotrimeric viral polymerases (PA, PB1, and PB2) (Compans et al., 1972; Zheng and Tao, 2013; Lo et al., 2018). After vRNPs are imported to the nucleus from the cytosol, vRNP acts as templates for mRNA transcription and complementary RNA (cRNA) replication. cRNPs and vRNPs are then selectively transported into the cytosol for new virion assembly (Pflug et al., 2017; Van Poelvoorde et al., 2020).

Hemagglutination protein, one of the major surface proteins, is a type I trans-membrane glycoprotein. The monomer HA molecule (HA0) consists of two subunits, namely, HA1 [327 amino acids (aa)] and HA2 (222 aa) located in the structure of a globular head domain on a stem domain. The HA globular domain includes the receptor-binding domain and vestigial esterase domain (only HA1). Stem domain is composed of HA2 and a component of HA1 (Wiley and Skehel, 1987). HA1 and HA2 change their conformation to mediate the fusion of viral membrane with the host membrane during the endosomal stage of the influenza life cycle (Steinhauer, 1999; Harrison, 2008; Sriwilaijaroen and Suzuki, 2012). The viral life cycle is mediated by HA protein, which is attached to sialic acid receptors. It also involves membrane fusion to release viral genome into cytoplasm and egress new progeny from infected cells (Chen et al., 2007; Brandenburg et al., 2013). Despite the fact that antigen mutation occurs at a high rate, HA-targeted influenza virus drugs have been researched and developed for many years, with a focus on the virus attachment and entry into cells, membrane fusion, viral release, and activation of HA0 into HA1 and HA2 (Corti et al., 2011; Ekiert et al., 2011; Dreyfus et al., 2012; Brandenburg et al., 2013; Yasugi et al., 2013). For instance, broadly neutralizing mAbs such as CR8033, CH65, 5 J8, and C08 bind to the receptor-binding domain of HA1 protein and block viral attachment. Others such as CR9114, CR6261, F10, CR8020, and FI6 bind to the stem domain inhibiting the membrane fusion steps. The antiviral antibodies also provide protection by engaging host effector cells through antibody-dependent cell-mediated cytotoxicity (ADCC) and complement-dependent cytotoxicity (CDC) (Chai et al., 2017; Chandler et al., 2023; Beukenhorst et al., 2024).

Meanwhile, an anti-DNA Abs, 3D8, was isolated from the spleen cells of MRL*-lpr/lpr* mice, an autoimmune prone mouse model that resembles human systemic lupus erythematosus (SLE). A recombinant 3D8 single-chain variable fragment (3D8 scFv) was produced as a combination of V_H_ (heavy chain variable single domain) and V_L_ (light chain variable single domain) (Kwon et al., 2002). 3D8 scFv with the ability to non-specifically hydrolyze both DNA and RNA and enter into the cytoplasm of the cell via caveolae/lipid raft endocytosis demonstrated a broadly antiviral effect against different viruses (Kim et al., 2006; Jun et al., 2010; Lee et al., 2014, 2021; Cho et al., 2015; Park et al., 2017; Hoang et al., 2022). An earlier report indicated that 3D8 scFv can hydrolyze messenger RNA (mRNA), viral genomic RNA (vRNA), and complementary RNA (cRNA) of influenza A viruses, resulting in a therapeutic effect *in vivo* (Lee et al., 2022).

Previous studies revealed that the double or triple combination of amantadine, ribavirin, and oseltamivir contributed to synergistic antiviral activity against a panel of influenza viruses (Nguyen et al., 2010). A synergistic antiviral effect against H1N1 PR8 IAV strain was achieved using nitazoxanide-oseltamivir combination treatment (Smee et al., 2013). The combined effect of oseltamivir and favipiravir accelerated clinical recovery from influenza infection but had little effect on the virus nucleotide diversity (Mu et al., 2023). These models provided a potential inhibition either targeting same viral proteins or two or more viral proteins and host and pathogen molecules which were reviewed in detail in a recent published literature (Batool et al., 2023). However, the emerging of drug-resistant variants of influenza viruses continues threaten the human health resulted in the urgent development of a novel antiviral drug.

In this study, a synergistic approach against influenza virus H1N1/PR8 *in vitro* was investigated. We successfully obtained a HA1-specific neutralizing antibody, NVLH8, using bio-panning. The single-domain V_L_ inhibited virus replication in a dose-dependent manner which was determined using plaque reduction and immunocytochemistry (ICC) assays. We found that NVLH8 neutralized virus infection at the viral genome releasing steps. The combination of 3D8 scFv, viral genome hydrolysis, and neutralizing antibodies created a synergistic effect against the influenza virus.

## 2 Materials and methods

### 2.1 Expression of HA1 antigen in yeast surface display

To express the antigen in YSD system, asparagine (Asn-N) was replaced with glutamine (Gln-Q) in six N-glycosylation sites in HA1 protein gene sequence of influenza virus A H1N1/PR8 (978 bp), (accession number EF467821.1) (N10Q, N11Q, N23Q, N268Q, N286Q, and N320Q) before synthesis. To amplify HA1 sequences, a specific primer pair conjugated with *NheI* and *BamHI* enzyme sites (forward, aaaGCTAGCGACACAATATGTATAGGCTAC, reverse, aaaGGATCCTCTGGATTGAATGGACGGC) was used in PCR. The HA1-PCR product was treated with enzymes *NheI* and *BamHI* (NEB, United States) and was introduced to empty pCTCON plasmid (ampicillin-resistant). Previously, *Saccharomyces cerevisiae* EBY100 was used as a model for YSD (Cho et al., 2020; Hoang et al., 2022). In brief, the plasmids were transformed to EYB100 using electroporation (BioRad, United States). EBY100 harbored the antigen plasmid, HA1::YSD, which was selected in SD media without tryptophan supplementation (Clontech, Japan). The antigen was expressed in SGCAA media containing 2% galactose. The HA1::YSD expression was analyzed in six different colonies by comparing with negative control (EBY100 only) using Western blotting and enzyme-linked immunosorbent assay (ELISA) with primary anti-cMyc antibodies (Invitrogen, Waltham, Massachusetts, United States). Overall, 20 μL yeast cells were used in Western blotting, while the yeast dilution at an OD of 0.4–0.6 (100 μL/well) was added onto a maxibinding immunoplate (SPL Life Sciences, Republic of Korea). The HA1::YSD expression was validated three times in every batch of the experiment.

### 2.2 Bio-panning

We used a previously described bio-panning technique to isolate HA1-specific candidates from human scFv libraries (Hoang et al., 2022). In brief, HA1::YSD was used as a target antigen to perform bio-panning with phage display scFv libraries. The scFv libraries (Tomlinson I + J) were expressed on phage using XL1 *E. coli* cells (tetracycline*-*resistant) and M13K07 helper phage. To isolate candidate-specific HA1::YSD, bio-panning was used as previously described (Lee et al., 2007; Cho et al., 2020; Hoang et al., 2022). In brief, the phage-displayed scFv libraries in a blocking buffer (3% BSA in Tris-buffered saline containing 0.1% (v/v) Tween 20 (TBS-T)) were first added to a 96-well maxibinding immunoplate (SPL Life Sciences, Republic of Korea) coated with PBS as a first negative selection. After incubation for 2 h at room temperature (RT, 25°C), the supernatant (SPNT) with non-binding scFv phages was transferred to the negative plate (EBY100 coated plate). The plate was incubated overnight at 4°C. Continuously, the non-binding SPNT phages were transferred to positive plate (HA1::YSD-coated plates) for 2 h at RT. Following the washing steps, the binding scFv phages were eluted using 100 μL/well of 100 mM triethylamine solution. The phages were amplified and used for the next round of bio-panning. After three rounds of bio-panning, the screening of HA1-specific scFv (HA1::scFv) was completed.

### 2.3 Phage ELISA

Phage ELISA was used to measure the affinity of the candidates to the positive samples. XL-1 *E. coli* blue at an OD_600_ of 0.6 was infected with the HA1::scFv-isolated phages, which spread across the LB agar plates. The phage colonies were randomly selected and cultured in 96-well plates (SPL Life Sciences, Republic of Korea). The HA1::scFv was expressed on phages with the addition of M13K07 helper phage in 2TY growth medium with 0.1% glucose at 30°C overnight. The SPNT was collected and transferred to HA1::YSD-coated plate for 2 h at RT. After washing five times with TBS-T, the plate was incubated for 1 h at RT with a 1:1,000 dilution of anti-M13 HRP-conjugated antibodies (Sino biological, China). The plate was rinsed with TBS-T five times following the addition of TMB substrate solutions (GenDEPOT, United States). Before measuring at an absorbance of 450 nm, we added 1 M sulfuric acid to the plate.

### 2.4 Identification of complementarity determining regions and framework region

The selected HA1-specific candidates were identified by PCR with a specific primer pair, LMB3, 5′-CAGGAAACAGCTATGAC-3′, and pHEN, 5′-CTATGCGGCCCCATTCA-3′ using 2X premix (Takara, Japan). The clones with 900 or 400 bp bands were sequenced using Macrogen (Republic of Korea). IgBlast tool from NCBI was used to blast the sequence of the CDRs and FR of each candidate (Martin, 1996; Johnson and Wu, 2000).

### 2.5 Production of protein

All the proteins in this research were expressed as soluble in *E. coli*. The sequences were introduced into pIg20 vector with the *XmaI* and *NcoI* enzymes (NEB, United States) that contain protein A at their C terminal and PhoA leader signal peptide at their N terminal. The expressed plasmids were transformed to *E. coli* BL21 (DE3 pLysE). The cells were cultured in Luria–Bertani (LB) broth with 100 μg/mL ampicillin and 25 μg/mL chloramphenicol at 37°C till OD _600_ reached approximately 0.8. The protein expression was induced by adding isopropyl 1-thiol-b-D galactopyranoside (IPTG) at 1 mM final concentration for 18 h at 25°C. Cell culture SPNT was filtered before being loaded into a column filled with IgG sepharose 6 fast flow resin (GE Healthcare, Chicago, United States). Next, the resin was washed by five bed volumes of PBS and five bed volumes of 5 mM ammonium acetate (pH 5.5). Later, the proteins were eluted from the resin with 10 bed volumes of 0.1 M acetic acid (pH 3.4) and neutralized by 1 M Tris–HCl (pH 9). The proteins were concentrated using Amicon® Ultra-15 Centrifugal Filter Units, 10 kDa (Merck, New Jersey, United States) and exchanged with PBS buffer, pH 7.4. The protein concentration was measured at OD_280_ with each protein’s extinction coefficient. The protein purity was verified by being loaded on the SDS page with Coomassie blue staining or Western blotting using a 1:3,000 dilution of anti-6X His tag (Abcam, United Kingdom).

### 2.6 vRNP hydrolyzing activity test of 3D8 scFv

To obtain vRNPs directly from influenza virions, the virus (1 × 10^5^ PFU) stationed in TBS buffer was incubated at 50°C for 30 min or treated with 0.1% Triton X100 at room temperature. The mixture was then added to 1 μg of 3D8 scFv protein in the presence of Mg^2+^ for 1 h at 37°C; DW and BSA (1 μg) were used as negative controls. These samples were used as template for a one-step reverse transcription (RT)-PCR using SuPrimeScript RT-PCR premix (Genet Bio, Daejeon, Republic of Korea) to detect eight gene segments (*HA*, *NA*, *NP*, *M1*, *PB1*, *PB2*, *PA*, and *NS1*) of the influenza virus with a band length of approximately 500 bp by their specific primers (Supplementary Table S1), following the instructions of the kit, 50°C/15 min, 95°C/5 min, 40 cycles (95°C/20 s, 60°C/30 s, and 72°C/30 s), and 72°C/10 min. The PCR products were loaded to 1% agarose gel containing ethidium bromide.

### 2.7 Influenza virions ELISA

Influenza virions were diluted in PBS and coated onto an maxibinding immunoplate (SPL Life Sciences, Republic of Korea) at 4°C overnight (1 × 10^5^ plaque-forming unit PFU/well). The NVLH8 protein (100 μL/well) in a 2X serial dilution (starting from the 100 μg/well) was added to the plate after incubation with a blocking buffer (5% skim milk in TBS-T buffer) for 2 h at RT. Following an incubation at 37°C for 1 h, 100 μL anti-6X His tag antibodies at a 1:3,000 dilution (Abcam, United Kingdom) were added to the TBS-T-washed plate for 2 h at RT. The virions coated in the plate were validated using polyclonal rabbit anti-HA antibodies (1:3,000 dilution; Invitrogen, United States). Goat anti-mouse IgG H&L (HRP) (1:5,000 dilution, Abcam, United Kingdom) and goat anti-rabbit IgG-HRP-conjugated antibodies (1:5,000 dilution, Invitrogen, United States) were added and incubated for 2 h at RT. After adding TMB and 1 M sulfuric acid, the plate was ready at OD450. The experiment was reproduced three different times independently. Each batch was designed with five data points, and three data points were chosen to be analyzed.

### 2.8 Influenza virus plaque assay

To titer the virus and evaluate the antiviral activity, we performed a plaque assay. In brief, MDCK cells were grown in 1 × 10^6^ cells/well in six-well plates (SPL Life Sciences, Republic of Korea) to fully confluence in Eagle’s minimal essential medium (Hyclone, United States) supplemented with 10% fetal bovine serum (Gibco, United States) and 0.1% antibiotic-antimycotic (Thermo Fisher Scientific, United States). The cells were incubated with the influenza virus for 1 h at 37°C. For the neutralizing test, serial dilutions of NVLH8 protein (0, 0.1, 1, 10, and 100 μg/mL) were pre-mixed with H1N1/PR8 viruses for 24 h at 37°C before the incubation step. The plate was overlaid with 1% Seaplaque agarose containing 1 μg/mL TPCK-trypsin in DMEM after withdrawing the inoculum. The plaques were stained with 0.5% crystal violet and counted after 3 days of incubation at 37°C. The assay was performed three times with different batches of protein expression. The data were expressed as the percentage of PFUs.

### 2.9 Influenza antiviral activity analysis

To test neutralization, influenza virus A H1N1/PR8, NVLH8 (100 μg/mL) was incubated with the viruses for 24 h at 37°C. The mixture was then incubated in MDCK cells (MOI 0.1) for 1 h at 37°C. After the removal of protein/virus complexes, the cells were cultured in virus growth medium [MEM-free medium supplemented with 0.2% BSA and TPCK-treated trypsin (1 μg/mL)]. The cells were harvested after 2, 4, 6, 8, 12, and 24 h of virus challenge and stored at −20°C for further RNA extraction.

To investigate the antiviral activity of 3D8 scFv post-treatment, the MDCK cells infected with H1N1/PR8 (MOI 0.1) were cultured in virus growth medium containing 3 μM of 3D8 scFv.

### 2.10 Immunocytochemistry

The neutralization activity of NVLH8 was analyzed using ICC. The ICC assay was conducted as described in a previous report (Hoang et al., 2022). In brief, following a neutralization assay of 24 h post-virus/antibody challenge to MDCK cells in 8-well chamber slides (SPL Life Sciences, Republic of Korea), the slides were rinsed with PBS. Ice-cold methanol was added for 15 min before the cells were permeabilized using Intracellular Staining Perm Wash Buffer (Biolegend, United States). After the cells were washed, a blocking buffer (1% BSA and glycine in PBST buffer) was added for 1 h. Polyclonal rabbit anti-HA antibodies at a 1:1,000 dilution (Invitrogen, United States) were added to detect the influenza viral HA protein, followed by an incubation with goat anti-rabbit IgG Alexa fluor 647 (1:1,000 dilution; Abcam, Cambridge, United Kingdom). The signals in the cells were visualized using a Zeiss LSM 900 confocal microscope with a 40X objective. The nucleus was stained with DAPI (LSbio, United States). The relative intensity percentage was calculated by dividing HA protein intensity (red signal) by DAPI signal (blue signal) in the same image and referenced to untreated samples (100%) with three different images.

Immunocytochemistry was also used to visualize the localization of neutralizing Abs. in cells. NVLH8 protein was premixed with H1N1/PR8 (MOI 10) at 300 μg/mL for 24 h at 37°C, and the protein at 300 μg/mL was used as a control (without the virus incubation). The virus/protein mixture was incubated with A549 cells for 1 h at 37°C and replaced with RPMI medium supplemented with BSA 0.2% and TPCK-trypsin 1 μg/mL. After 4 h of incubation, the cells were processed using the same method but with different antibodies. The cells were incubated with mouse monoclonal anti protein A antibodies (1:1,000; Sigma, United States) and goat anti-mouse IgG Alexa fluor 488 (1:1,000 dilution; Abcam, Cambridge, United Kingdom).

To evaluate the penetration of 3D8 scFv protein into cytoplasm using ICC, we treated MDCK cells with 3D8 scFv (5 μM) for 0.5, 1, 3, 6, 12, 24, and 48 h. The protein was detected using 3D8-specific antibodies (Abclon, #3B3, Incheon, Republic of Korea) at 1:1,000 dilution and goat anti-mouse IgG Alexa fluor 488 (1:1,000 dilution; Abcam, Cambridge, United Kingdom).

### 2.11 Hemagglutination titer and hemagglutination-inhibition assay

Hemagglutination (HA) assay was carried out to titer the influenza A viruses using HA units on chicken red blood cells (cRBCs) (Innovative Research Inc., Novi, MI, United States), as described previously (Kaufmann et al., 2017). Overall, 50 μL of cRBCs diluted to 1% PBS was distributed into 2-fold dilution of 25 μL viruses in 25 μL PBS followed by an incubation at room temperature for 1 h. The lowest virus titers that inhibit cRBC precipitation were determined at 1 HA units. Two further dilutions of the HA titration were defined as 4-fold HA units (4 HA) which were used for hemagglutination-inhibition (HI) assay.

For the HI assay, to determine inhibition activity of specific antibodies to HA antigen, 4 HA of 25 μL of influenza viruses were added to 25 μL of 2-fold serial dilution of five proteins, started at 1,000 μg/mL of NVLH8 in an immunology plate (SPL Life Sciences, Pocheon-si, Republic of Korea), followed by an incubation at RT for 1 h. After the addition of 50 μL of 1% cRBCs, the plate was incubated for 1 h/RT. The HI titer was determined at the lowest amount of protein (μg/mL) that inhibited hemagglutination by direct visualization.

### 2.12 Synergistic test of neutralizing NVLH8 and 3D8 scFv

Influenza virus A H1N1/PR8 at 2 × 10^4^ PFU/mL was neutralized using 100 μg of NVLH8 for 24 h at 37°C. The mixture was then incubated with MDCK cells (MOI 0.2) for 1 h at 37°C. Followed by the removal of the infection medium, the medium was changed to virus growth medium with 3D8 scFv (3 μM) and then incubated at 37°C with 5% CO_2_ for 18 h. Single treatment either with NVLH8 or 3D8 scFv alone was conducted at the same time as non-treated sample. The cells or SPNT were collected and stored at −20°C for further analysis. While the cells were used for RNA extraction and protein extraction with RTqPCR and Western blotting, respectively, the SPNT was subjected to a plaque assay. The protein was harvested using RIPA buffer to lyse the cells, according to the manufacturer’s protocol (Biosolution, Republic of Korea). Western blotting was performed with 20 μg of protein from each sample using influenza A M2 polyclonal antibodies, polyclonal anti-HA antibodies (1:5,000 dilution, Invitrogen, United States), and monoclonal anti-GAPDH antibodies (1:100 dilution, Santa Cruz Biotechnology, United States) as the primary antibodies.

### 2.13 Quantitative real-time PCR and one-step RTqPCR

Total RNA was extracted from the harvested cells using the TRI reagent (MRC, United States) with chloroform and iso-propanol method. To measure the vRNA, cRNA, and mRNA of influenza, strand-specific qPCR was conducted. cDNAs specific to vRNA, cRNA, or mRNA of *HA* and *NP* genes were synthesized by specific primers using superscript IV first strand synthesis system (Thermo Fisher Scientific, USA), as shown in Supplementary Table S2, following the manufacturer’s protocol. The cDNAs were used as a template for qPCR using SYBR Premix Ex Taq and the Rotor-Gene Q system with the strand-specific primers to *HA* and *NP* genes (Supplementary Table S3). Data were analyzed using Rotor-Gene Q series software version 2.3.1. On the other hand, one-step RT-qPCR was performed on the extracted RNA using Accupower GreeenStar RT-qPCR Premix and Master Mix (Bioneer, Republic of Korea) and Rotor-Gene Q System (Qiagen) to measure the antiviral effect of the candidates. The relative expression level of each RNA type in 3D8 scFv-treated groups was normalized to that of the untreated groups (H1N1/PR8) (calibrated as 1). The primers are shown in Supplementary Table S4. *GAPDH* was amplified as an internal control and used for relative expression analysis.

### 2.14 Statistical analysis

The figures were presented in GraphPad Prism 8.0 software (GraphPad Software, United States). Statistical significance (asterisks) was determined using a one-way ANOVA, unpaired *t*-test, (ns: non-significant, ^*^p < 0.05, ^**^p < 0.001, ^***^p < 0.0005, and ^****^p < 0.0001). Data are shown as the mean with standard deviation (SD) of triplicate samples.

## 3 Results

### 3.1 Screening of HA1-specific antibodies

**Figure 1:**
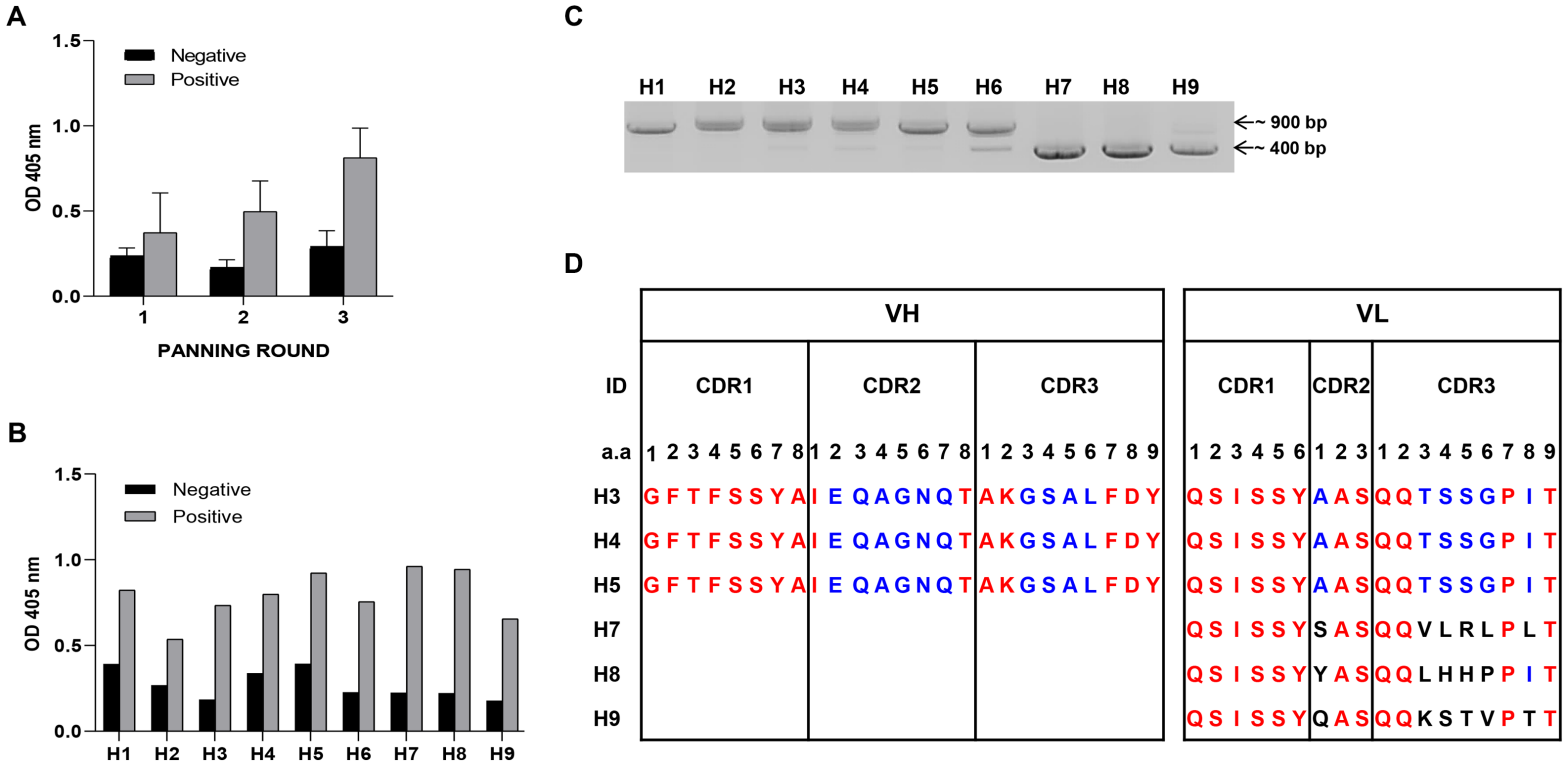
Selection of HA1 specific candidates **(A)** Bio-panning was used to screen HA1 specific candidates. Three rounds of bio-panning were performed with HA1: YSD as positive samples and EBY100 as negative samples using Phage Elisa. Data are expressed as means ± SD. **(B)** From a hundred random phagemid colonies, nine colonies that showed highest binding affinity to HA1: YSD when compared with negative samples were isolated. **(C)** Detection of the nine candidates, either in scFv (900 bp) or single-domain forms (400 bp) with specific primers using PCR. **(D)** Alignment of CDR regions of each single-domain of candidates.

To optimize antigen expression in YSD, the removal of N-glycosylation sites in the sequences is required. Six N-glycosylation sites in HA1 protein gene sequence of influenza virus A H1N1/PR8 (978 bp) were changed from asparagine to glutamine (N to Q) (Supplementary Figure S1A). The amplified HA1-DNA fragment was cloned into an YSD expression vector (Supplementary Figures S1B,C). Six different yeast colonies expressed HA1 antigen successfully in contrast with the negative control (EBY100), which was confirmed using Western blotting and ELISA (Supplementary Figures S1D,E). Three rounds of bio-panning were performed, with the antigen binding affinities of scFv candidates to positive samples (HA1::YSD) increasing with each round, compared with negative controls (EBY100 yeast) (Figure 1A). Continuously, the nine clones that showed the highest affinity to HA1::YSD by phage ELISA were isolated (Figure 1B). Detection of the clones using PCR with specific primers indicated that these candidates appeared in different forms, either in scFv (900 bp) or in single-domain V_H_ or V_L_ (400 bp) (Figure 1C). The CDRs and FRs of the candidates were identified using the IgBlast Kabat antibody sequence database (Ye et al., 2013). Several stop codons existed in HA1-specific scFv (H1, H2, and H6) in CDRs and FRs (data not shown). Interestingly, H3, H4, and H5 scFv candidates shared the same amino acid sequences without any stop codons in CDRs and FRs, indicating the high frequency of the candidates and the diversity of the libraries. Three single-domain clones (H7, H8, and H9) with 400 bp sizes belonged to the VL sequences and shared differences in the first amino acid at CDR2 and a few amino acids at CDR3 (Figure 1D).

**Figure 2:**
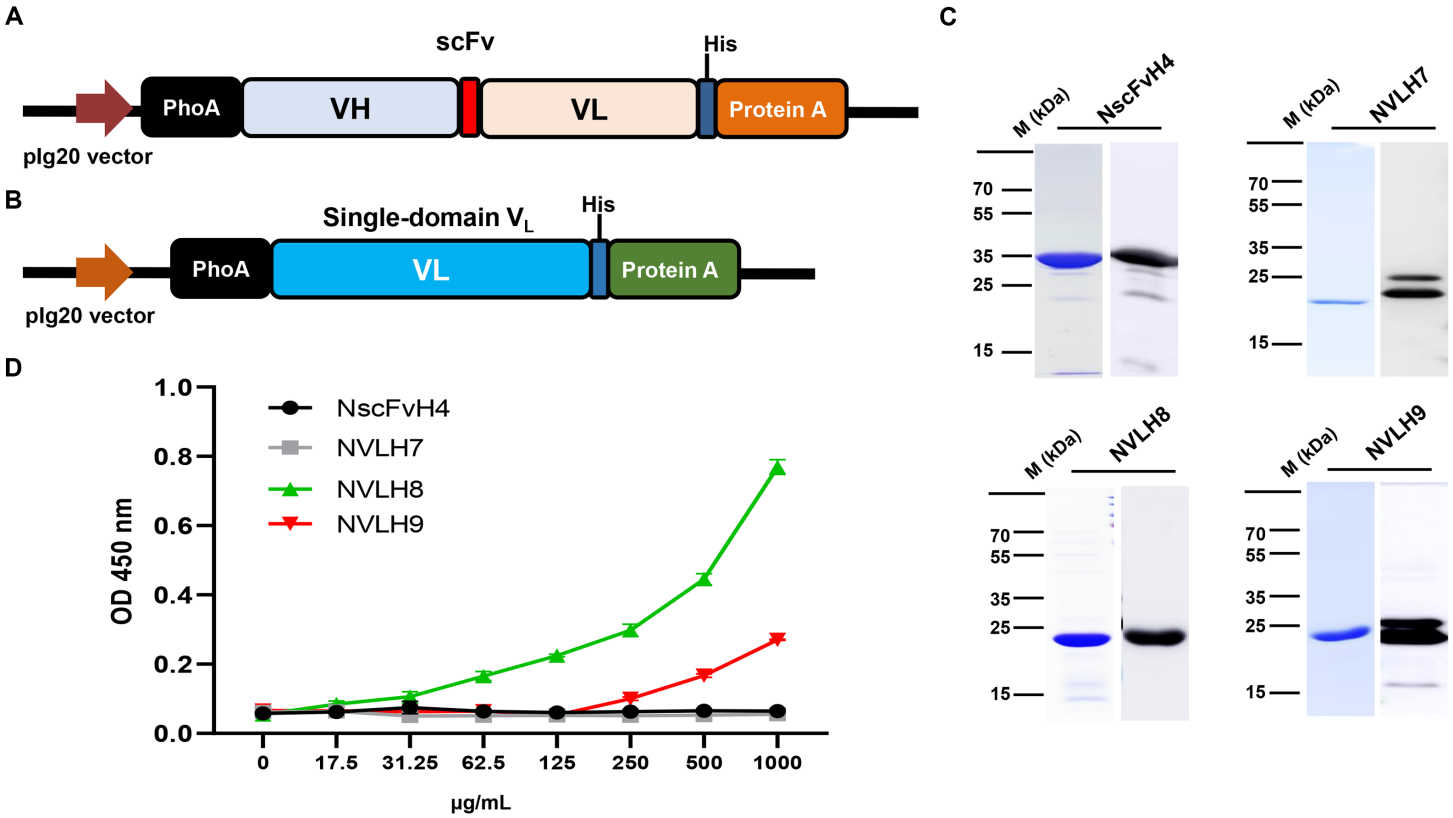
Purification and characterization of chosen HA1 candidates in *E. coli*. The HA1 candidates were introduced into the expression pIg20 vector containing a PhoA signal peptide at the N terminal and fused with protein A to improve purification. Expression vector of scFv (NscFvH4) **(A)** and three different single-domain V_L_ candidates (NVLH7, NVLH8, and NVLH9) **(B)**. **(C)** Purified proteins were confirmed by Coomassie blue staining and Western blotting using anti-His-Abs, from left to right, above NscFvH4; NVLH7; bottom NVLH8 and NVLH9. **(D)** Virion binding affinities of the four purified candidates using virion ELISA in a concentration-dependent manner. Error bars indicate mean with SD of triplicate samples. The background was subtracted.

Because H3, H4, and H5 scFv candidates had identified sequences, one of them, H4 (renamed as NscFvH4), was picked to be produced at a protein level together with three different single-domain V_L_, NVLH7, NVLH8, and NVLH9. These were cloned into pIg20 vector containing PhoA signal peptide and protein A (Figures 2A,B). The proteins were expressed in soluble form in *E. coli* and purified using IgG sepharose. The purified proteins were verified using Coomassie blue staining and Western blotting with anti-6X His tag antibodies sized 30.1 kDa for NscFvH4, and 22.5 kDa for NVLH7, NVLH8, and NVLH9 (Figure 2C). Each protein was obtained in a different yield and purity (Table 1). Although NscFvH4 and NVLH8 had similar purity (88 vs. 87%, respectively), NVLH8 was obtained at 3 mg/L, three times greater than NscFvH4, at 1 mg/L. In contrast, NVLH7 and NVLH9 were obtained at a low yield (0.3 and 0.5 mg/L, respectively) and at 70% purity despite similar constructs as NVLH8 (Figure 2D). The function of recombinant proteins was assessed using virion ELISA with a 2X serial dilution for direct antigen binding affinity to the influenza virus particle H1N1/PR8. Among the candidates, in a concentration-dependent manner, NVLH8 demonstrated the highest binding strength to virus H1N1/PR8 particles. NVLH9 demonstrated a weak interaction with virus particles, whereas NscFvH4 and NVLH7 did not demonstrate the same (Figure 2D).

**Table 1:** Characterization of expressed candidates with purification yield and purity.

Name	Form	Size (kDa)	Expression	Yield (mg/L)	Purity (%)
NscFvH4	V_H_-L-V_L_	33.4	Secret	1	88
NVLH7	V_L_	22.6	Secret	0.3	71
NVLH8	V_L_	22.7	Secret	3	87
NVLH9	V_L_	22.7	Secret	0.5	70

### 3.2 Single-domain V_L_ NVLH8 neutralized specifically to H1N1/PR8 influenza infection through preventing viral genome from releasing into the cytoplasm

**Figure 3:**
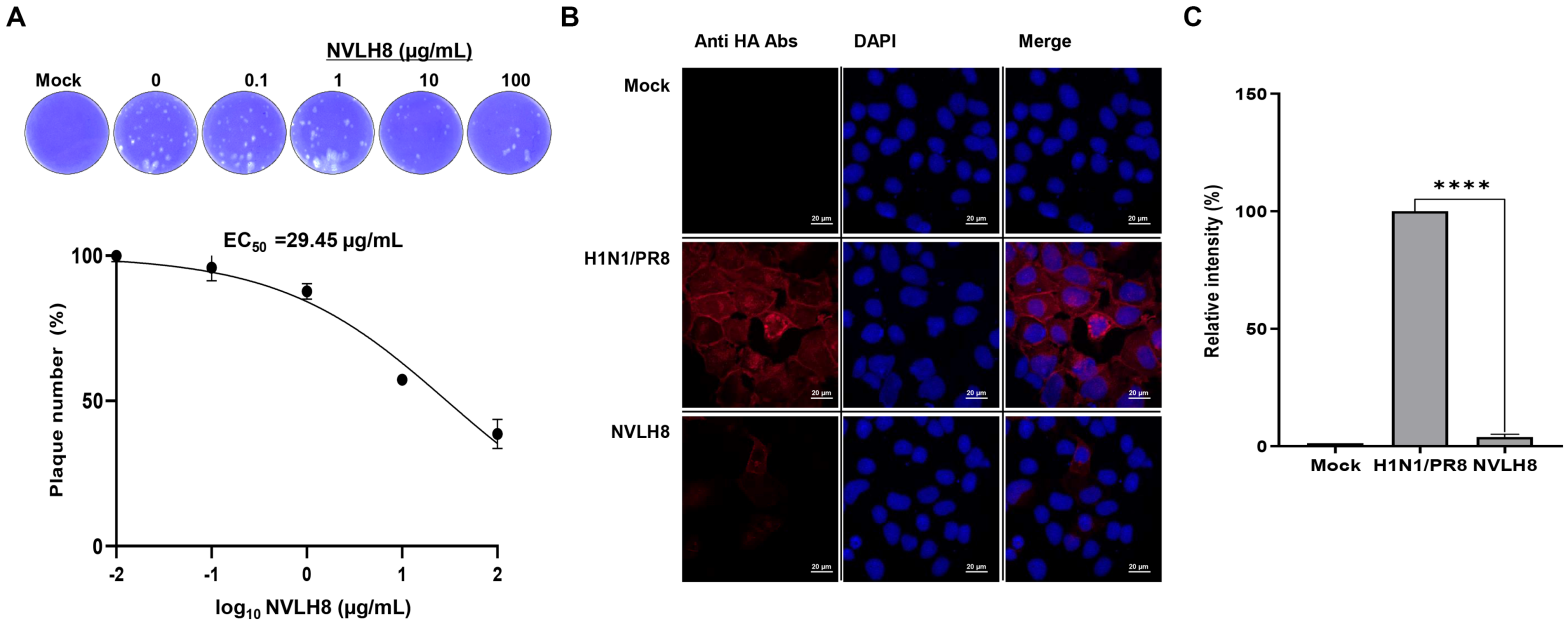
Single-domain V_L_ (NVLH8) neutralized influenza virus *in vitro*. **(A)** Infection of influenza H1N1/PR8 virus in MDCK cells was prevented in a treatment of NVLH8 serially diluted concentrations (0, 0.1, 1, 10, and 100 μg/mL) through plaque inhibition assay in three independent repeats, error bars indicate SEM. The results of the plaque inhibition assay were expressed as percentages. The EC50 curve fitting was obtained using GraphPad Prism 8 from the plaque inhibition assay data. **(B)** Neutralization activity of NVLH8 (100 μg/mL) caused reduction of viral protein expression (HA protein) in MDCK cells through ICC (magnification 40X). **(C)** The intensity of viral protein [from **(B)**] was converted to relative intensity percentages by normalizing to DAPI intensity. Error bars indicate mean with SD of triplicate samples.

To test whether NVLH8 neutralized virus infection, we performed a plaque inhibition assay. The protein caused a decrease in plaque number in a concentration-dependent manner. Specifically, NVLH8 at 0.1, 1, 10, and 100 μg/mL neutralized viruses with reductions of 96, 88, 57, and 38%, respectively, showing the EC50 value of 29.45 μg/mL (Figure 3A; Supplementary Figure S4). Moreover, the viral HA protein signal (red) in samples neutralized with 100 μg of single-domain NVLH8 was not visible when compared with positive samples using ICC (Figure 3B). The HA protein intensity decreased significantly in the presence of the protein (Figure 3C; Supplementary Figure S5).

**Figure 4:**
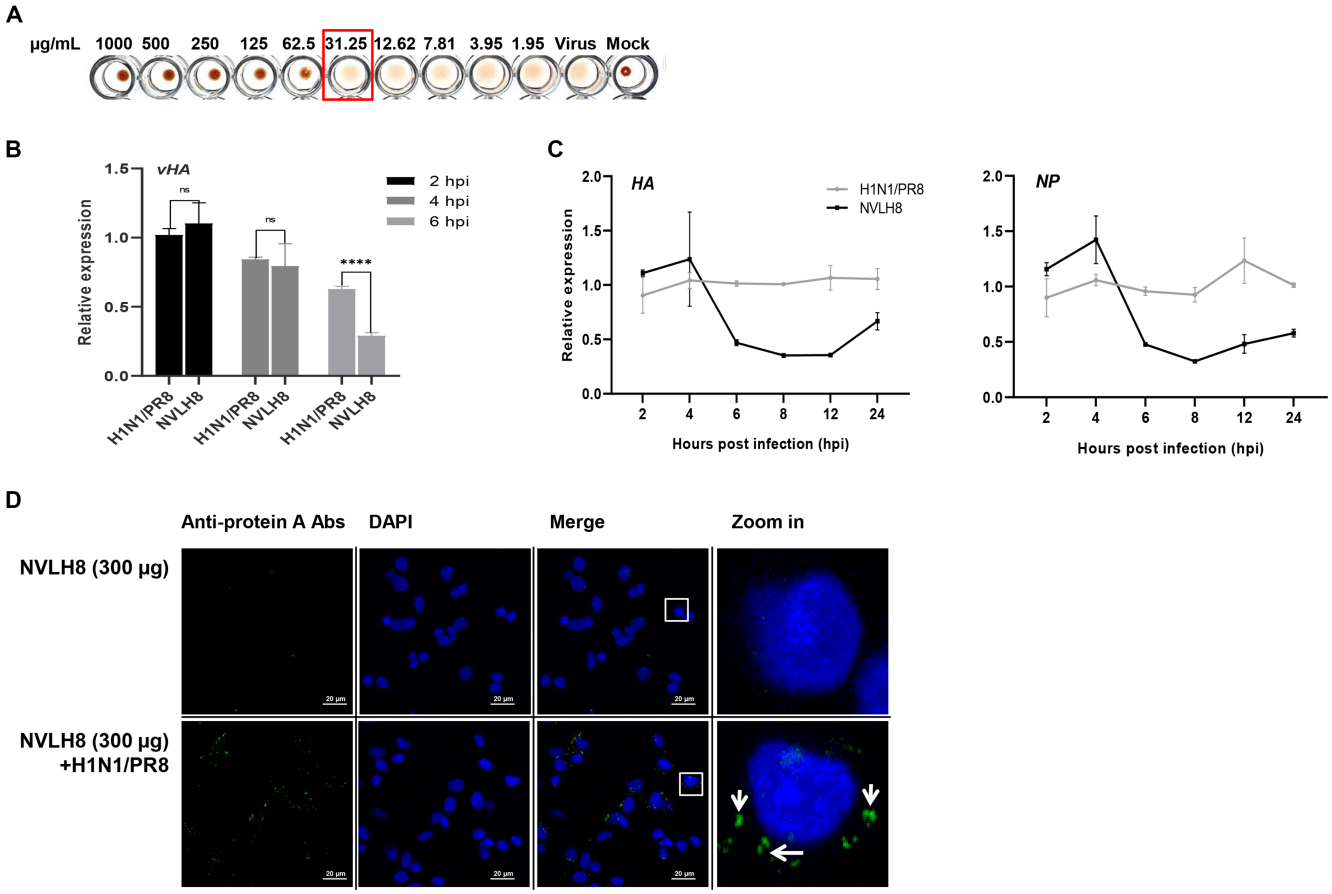
Inhibition of influenza viral genome releasing into cytoplasm by internalized into cells of virus bound NVLH8 protein. **(A)** HI activity of the NVLH8 to 4-fold HA units of H1N1/PR8 in the presence of 1% cRBCs. **(B)** H1N1/PR8-HA-viral RNA (vHA) detection at 2, 4, and 6 hpi in the treatment of NVLH8. **(C)** The neutralization activity of NVLH8 was compared at gene level of the viral genome (HA), (NP) in a time-dependent manner. The protein blocked the viral gene releasing steps rather than the entry steps. **(D)** The virus-bound NVLH8 protein was internalized into cells. A549 cells were inoculated either with 300 μg/mL of the protein (NVLH8) or a 24 h/37°C premixed of H1N1/PR8 (MOI 10) and 300 μg/mL of the protein. Following, the cells were cultured for 3 h more. The proteins were detected using the primary monoclonal mouse anti-protein A antibodies. Goat anti-mouse IgG Alexa 488 (green) antibodies were used as secondary antibodies. The nuclei were stained with DAPI (blue). White squares in merge panels were enlarged to show the protein inside of the cells (green) with a white arrow. Statistical significance was determined using the unpaired *t* test (ns: non-significant, ^****^p < 0.0001), error bars indicate SD of triplicate samples.

The neutralizing NVLH8 exhibited binding affinity to virus particles (Figure 2D), resulting in virus inhibition (Figure 3). Therefore, we studied the neutralization mechanism of the single-domain V_L_ NVLH8. First, the protein exhibited HI activity at 31.25 μg/mL (Figure 4A). The findings indicated that NVLH8 had affinity for the HA1 subunit at a receptor binding site, which may prevent the viral binding to cell-surface receptors. Since the protein demonstrated a consistent relationship between the HI activity and the neutralization efficacy via plaque reduction, we hypothesized two possible neutralization mechanisms of the protein: (a) it inhibits the viral attachment to receptors or (b) it prevents the un-coating steps during viral entry by interfering with membrane fusion.

To figure out whether NVLH8 inhibits the virus entry or viral gene releasing steps, we quantified the vRNA (*vHA*) number of H1N1/PR8 levels in the infected cells in the early stages (2, 4, and 6 h post infection; hpi) (Figure 4B). At 2 and 4 hpi, there were no differences in HA vRNA levels between the NVLH8 treatment and the control (H1N1/PR8). The protein’s inhibitory activity was clearly demonstrated at 6 hpi by 45% reduction in *vHA* level. We then used NVLH8 to observe the time course of *HA* and *NP* expression in neutralized virus infection (Figure 4C). The *HA* and *NP* viral genomes decreased from 6 to 8–12 hpi. It is suggested that the viruses which were not inhibited by NVLH8 were able to replicate normally, which is why at 24 hpi, the *HA* and *NP* levels were slightly higher than at 12 hpi.

The results supported the hypothesis that the candidates inhibited the viral genome releasing steps, implying the blockage of membrane fusion steps rather than the entry steps. To further validate this, the virion binding proteins internalized with the virus were postulated. We tested the protein NVLH8 localization in the cells in the presence of viruses. To better visualize the protein, protein NVLH8 (300 μg/mL) was pre-incubated with H1N1/PR8 at MOI 10 for 24 h before its incubation with the cells. In addition, to prevent the degradation of the protein, the cells were fixed after 4 hpi. Using immunofluorescence staining, the bound NVLH8 (green) was found at 300 μg/mL dose in the presence of the viruses compared with no virus-treated samples (NVLH8) (Figure 4D). The finding that the Abs localized in the cells after being premixed with the viruses suggested that such a candidate prevented viral infection by inhibiting viral genome release.

### 3.3 Influenza antiviral synergistic effects in a combination with 3D8 scFv of neutralizing NVLH8

We first evaluated the antiviral activity of 3D8 scFv after viral infection. The penetration of 3D8 scFv into MDCK cells was investigated. 3D8 scFv protein (green) was taken up at a very early time (0.5 h) and peaked at 6–12 h and remained in the cytoplasm for up to 48 h (Supplementary Figure S2A). Subsequently, the antiviral activity of 3D8 scFv post-treatment against H1N1/PR8 was evaluated in post-viral infection through the decrease of viral RNA levels of *HA*, *M1*, *NP,* and virus titer (Supplementary Figures S2B,C).

**Figure 5:**
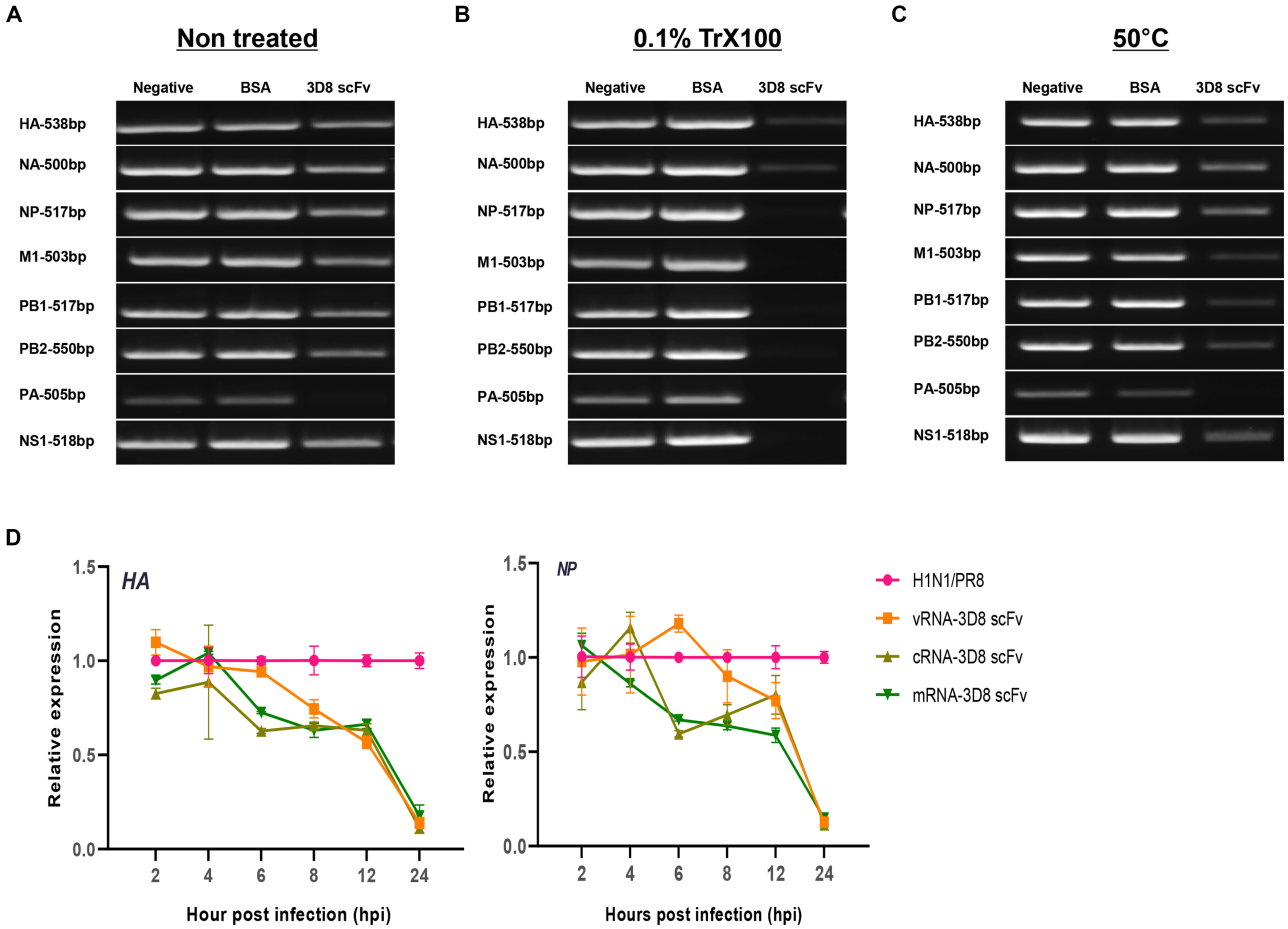
3D8 scFv hydrolyzed all types of RNA of influenza viruses (H1N1/PR8). Virus particles **(A)** without treatment with any chemicals or temperature, **(B)** treated with 0.1% Trx100 at room temperature to release vRNP in the TBS buffer, **(C)** incubated at 50°C/30 min with 3D8 scFv (1 μg) in presence of Mg^2+^ at 37°C/1 h. These templates were used for RT-PCR with eight set primers of the H1N1/PR8 gene with band size around 500 bp. For each set of primers (HAvRNA, HAcRNA, HAmRNA, NPvRNA, NPcRNA, and NPmRNA), RNA samples collected over time in a 3D8 scFv post-viral infection treatment were used to synthesize cRNA, and used as template for qPCR with specific primer sets. **(D)** Three types of RNA of *HA* and *NP* genes over time course. Data were reproduced triplicate and expressed as mean with SD.

**Figure 6:**
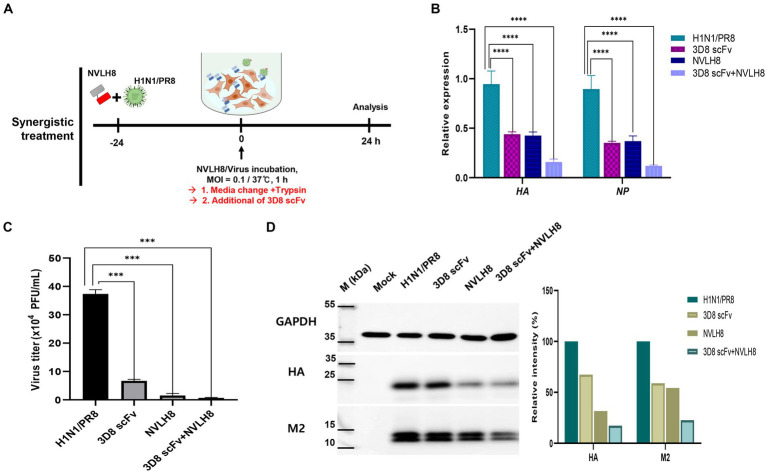
Synergistic effect of neutralizing antibody as a vertical effect and catalytic antibodies 3D8 scFv as a horizontal effect. **(A)** Scheme of synergistic treatment. H1N1/PR8 were premixed with 100 μg of NVLH8 for 24 h and incubated with MDCK cells for 1 h/37°C. After 24 hpi, the cells were harvested to measure the synergistic affect. **(B)** RTqPCR with the relative expression of viral gene (*HA* and *NP*) was conducted to compare between un-treated group (H1N1/PR8),the single treatment group (3D8 scFv or NVLH8), and the combination of 3D8 scFv and NVLH8 treatment group, **(C)** plaque reduction assay and **(D)** intracellular protein Western blotting with anti GAPDH, HA, and M2 protein from. Statistical significance was determined using the unpaired *t* test (ns: non-significant, ^*^p < 0.05, ^**^p < 0.001, ^***^p < 0.0005, and ^****^p < 0.0001). Data are shown as mean with SD of triplicate samples.

Our previous study reported the antiviral activity of 3D8 scFv against influenza H1N1/H275Y virus through hydrolyzing viral RNA in mRNA and vRNPs and cRNPs form (Lee et al., 2022). Here, we strongly agreed that 3D8 scFv targeted not only mRNA (naked RNA) but also vRNA and cRNA in RNP forms (vRNPs or cRNPs) of H1N1/PR8. Most of those bands were invisible or blurry in 3D8 scFv-treated samples when compared with negative controls (DW) or BSA (Figure 5B). However, those bands were detected in samples without non-0.1% Triton X100 even in the presence of 3D8 scFv (Figure 5A). The thickness of those 3D8 scFv-treated bands was less than that of the control sample bands. During the reverse transcription step at 50°C, vRNPs were assumed to be partially discharged, and 3D8 scFv, which was stable at high temperature, had bound and cleaved the free vRNPs. Consequently, the virus particles were incubated at 50°C for 30 min. The heating process could also release vRNPs that were digested by 3D8 scFv. However, the vRNPs were not released as much as in the 0.1% Triton X100-treated condition (Figure 5C). A post-viral infection at various times with 3D8 scFv post-treatment was conducted to analyze the changes in each type of RNA. At specific time points during virus replication, the expression of the three RNAs of the *HA* and *NP* segments was measured (Figure 5D). Particularly, reductions were observed clearly at 12 hpi (~30–40%) and 24 hpi (80%) for all three types of RNAs of *HA* and *NP* segments (Figure 6D) but not at earlier time points. The data suggested that 12 hpi was critical for 3D8 scFv antiviral activity when 3D8 scFv had fully penetrated and was released into the cytoplasm to bind and digest the three viral RNAs.

A synergistic efficacy of NVLH8 neutralizing Abs (i.e., targeting early stages) and 3D8 scFv (intermediate and late stage) was investigated. A combination treatment (Figure 6A) in which the neutralized H1N1/PR8 virus with NVLH8 was infected into MDCK cells and then treated with 3D8 scFv in virus growth medium was used. The antiviral activity of a combination treatment (3D8 scFv and single neutralizing Abs) was compared with that of a single treatment using intracellular viral gene expression (HA and NP) (Figure 6B). Individually, while 3D8 scFv caused relative viral gene reduction of approximately 56% (*HA*) and 65% (*NP*), neutralizing NVLH8 decreased *HA* and *NP* gene by 57 and 63%, respectively, and a combination treatment reduced *HA* and *NP* by 84 and 88%, respectively. These results suggested that combining a viral genome hydrolyzing 3D8 scFv with an influenza neutralizing Abs amplified the antiviral efficacy. The synergistic efficacy was consistently demonstrated in the reduction of virus titer using plaque assay (Figure 6C, Supplementary Figure S6) and viral protein using Western blotting (Figure 6D).

**Figure 7:**
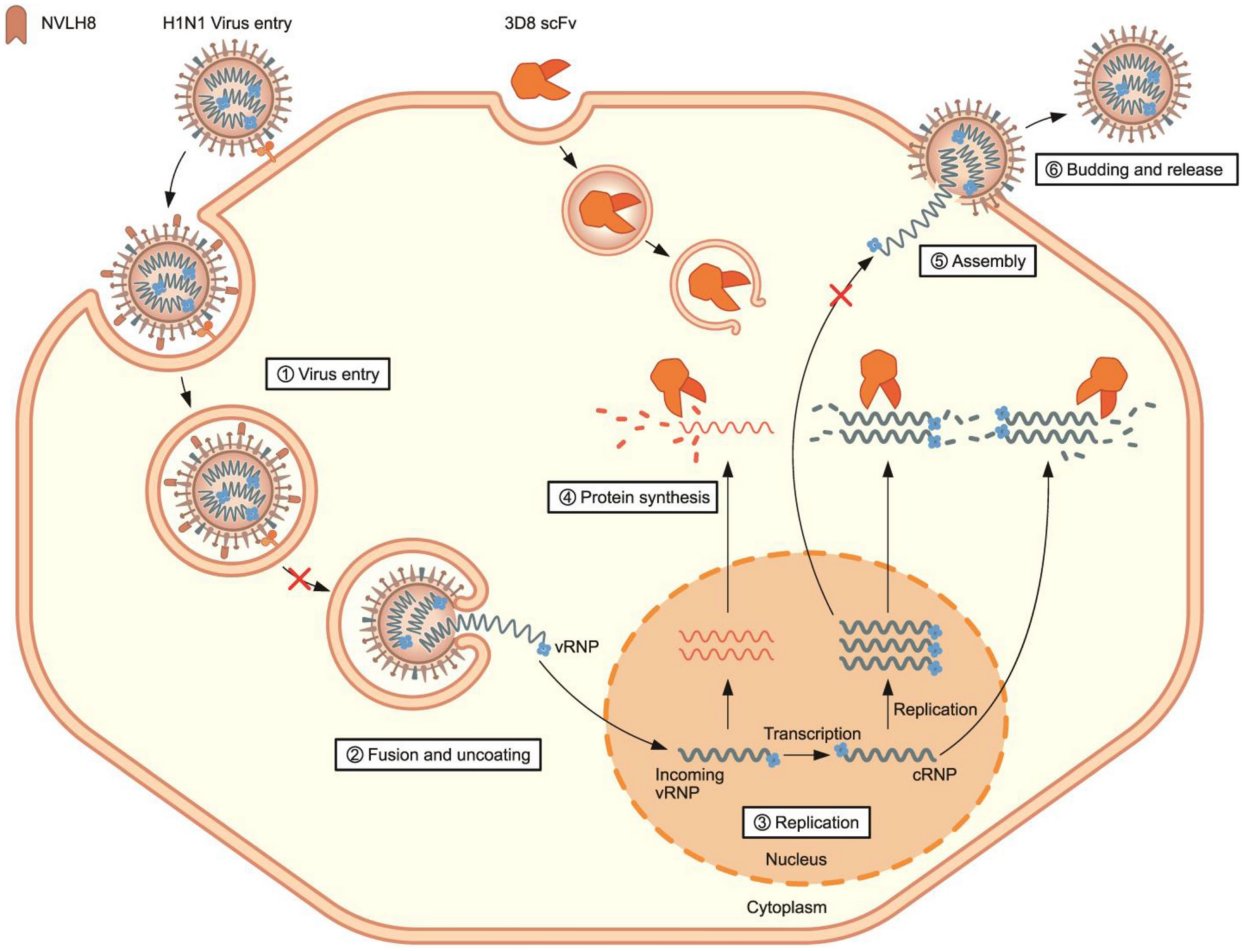
Proposed model of synergistic antiviral mechanism of neutralizing NVLH8 and 3D8 scFv. The synergistic antiviral activity can be achieved using two different antibodies with two different mechanisms of action. H1N1/PR8 virus carried neutralizing NVLH8 protein and entered into cells. The bound NVLH8 blocked the virus releasing vRNPs into cytoplasm. On the other hand, the presence of 3D8 scFv target viral genomes at intermediate and late stages in the virus’s life cycle.

Therefore, a synergistic effect of a combination treatment of different therapeutic targets to different stages of influenza viral life cycles is proposed (Figure 7). NVLH8 neutralizing Abs bound to the influenza virus, internalized together into cells, and prevented the viral genome releasing steps, whereas 3D8 scFv localized in the cell cytoplasm by caveolae-mediated endocytosis and degraded viral genome in the intermediate and late stages of the life cycle of the virus. A synergistic effect can be achieved by using neutralized Abs-specific H1N1/PR8 as a vertical effect at viral entry steps, the action of 3D8 scFv hydrolyzing all types of IAV RNAs/RNP at viral protein biosynthesis, and exclusion of virus from the cytoplasm of infected cells as a horizontal effect.

## 4 Discussion

All influenza antivirals in the market or in development mainly target influenza proteins, such as M protein (M2 ion channel blocker), NA protein (NA inhibitor), polymerase proteins (RdRP inhibitor, cap-dependent endonuclease inhibitor, polymerase inhibitor, PB2 inhibitor, PB1 inhibitor, and PA inhibitor), HA protein (HA fusion inhibitor, HA maturation inhibitor), NP protein (NP inhibitor), or host-targeted recombinant sialidase fusion protein (De Clercq, 2006; Byrn et al., 2015; Naesens et al., 2016; Yen, 2016; Noshi et al., 2018; Van Poelvoorde et al., 2020; Jones et al., 2023; Kumari et al., 2023; Zhang et al., 2024). Development of HA-targeted influenza virus drugs mainly focus on several mechanisms: (1) receptor-binding pocket (RBP) to prevent the virus attachment and entry to cell, (2) esterase domain or HA2 to block the membrane fusion, (3) globular head to inhibit the viral releases, and (4) stalk domain to prevent cleavage of HA0 into HA1 and HA2 (Corti et al., 2011; Ekiert et al., 2011; Dreyfus et al., 2012; Brandenburg et al., 2013; Yasugi et al., 2013; Finney et al., 2024). The anti-influenza neutralizing Abs directed to HA globular domain contains receptor binding site (RBS), which block virus attachment to host cell (Yoshida et al., 2009) or the stem domain, inhibiting membrane fusion (Okuno et al., 1993). Normally, neutralizing antibodies bound to RBS are often strain-specific. Binding to stem domains, on the other hand, is a broadly neutralizing activity to various flu viruses but less effective in neutralization (Lee et al., 2019).

Since the successful isolation of monoclonal antibodies (mAbs) by the hybridoma method in 1975, antibody therapies have been widely used in the prevention and treatment of viral infections (Köhler and Milstein, 1975). Therapeutic antibodies for viral infections were generated using several approaches: (1) phage display antibody libraries, (2) single memory B cells, (3) single antibody secreting plasma B cells, (4) proteomics-directed cloning of mAbs from serum, and (5) deep sequencing of paired antibodies encoding genes from B cells (Salazar et al., 2017).

In the present study, antigen HA1-specific candidates were isolated using bio-panning. First, the antigen, protein HA1, was displayed on the YSD (Supplementary Figure S1). YSD is a system with a wide range of applications in protein engineering (Boder et al., 2012; Gera et al., 2013). YSD can also resemble the HA1 protein on the virus surface, therefore enhancing specific selection. Bio-panning with phage ELISA method was applied to screen the target candidates, including scFv and single-domain forms (Figure 1). The candidates were expressed and purified as secreted forms in *E. coli* with varying yields and purities (Figure 2; Table 1). The chosen single-domain V_L_ (NVLH8) showed the strongest binding affinity to virus particles (Figure 2D), resulting in a neutralizing activity against H1N1/ *in vitro* (Figure 3). The differences in binding affinity between those candidates can be attributed to differences in a few amino acids in CDRs. It should be noted that the described neutralization assay is an unconventional method in that the virus/antibody must be incubated for 24 h rather than 1 h. Indeed, prior to performing this neutralization assay, we were unable to obtain neutralization activity using conventional methods (1 h of incubation). Following many trials, we demonstrated that the neutralizing activity was observed after 24 h of incubation. We compared many different neutralizing time points, particularly at 1 and 24 h, to ensure that the neutralizing activity was only visible for 24 h (Supplementary Figure S3A). We also attempted to confirm infectivity of the influenza virus to MDCK cells after 24 h of incubation. The virus was not affected, but the virus titer was reduced to half (data not shown). Several models have been proposed for neutralization antibodies. The model “occupancy or coating” is defined by obtaining a sufficient number of antibodies to interact with the surface of virions in order to block the viral attachment or fusion process, whereas the critical binding site model indicated that neutralization takes place when binding occurs not only to virions but also to specific binding sites (Burton et al., 2001; Marasco and Sui, 2007; Burton, 2023; Kumari et al., 2023). These models could be used in the development of therapeutic antibodies that target the most critical neutralization site with the highest affinity. Therefore, a critical binding site model should be used for the neutralization activity of NVLH8.

We further found that the neutralizing antibodies exhibited the HI activity to H1N1/PR8 (Figure 4A), suggesting binding to globular domain. Interestingly, binding to virions, particularly to HA protein (probably HA1 globular domain), did not affect virus attachment as the unchanged viral RNA (*vHA*) at 2 hpi. At 2 hpi, the virus enters into the host cell after attaching to the receptors. The NVLH8 probably inhibited the vRNP releasing steps by lowering vHA levels at 4 and 6 hpi (Figure 4B). The next 2 h (4 hpi) are required for membrane fusion to allow complete release of vRNA into the cytoplasm (6 hpi). Subsequently, reduction in the *HA* and *NP* genome levels was observed in a time course treatment in which the viral genome levels dropped dramatically at 8 hpi and slightly increased at 12 and 24 hpi (Figure 4C). Additionally, the NVLH8 detected inside the cells in the virus pre-mixture by ICC was proposed to inhibit the fusion process based on the interaction with HA subunit (Figure 4D). Furthermore, depending on the IAV strain, NVLH8 demonstrated the specific neutralization activity (H1N1/pdm, H3N2/Brisbane, and H3N2/Switzerland), which supported that they are specific to H1N1/PR8 (Supplementary Figure S3B).

In a previous study, a 3D8 scFv was able to hydrolyze RNA in RNP form, resulting in an antiviral activity against influenza viruses (Lee et al., 2022). Here, we strongly agree that the 3D8 scFv digested RNA in RNP form, particularly to H1N1/PR8 strain. We also emphasized that the 3D8 scFv post-treatment revealed a therapeutic effect on targets mRNA, vRNA, and cRNA during viral infection and replication in the host cells. The appearance of 3D8 scFv in the cytoplasm resulted in reduction of the viral gene cytoplasmic levels, mainly from the intermediate stage to late stage of the influenza A virus cycle (Figure 5).

The combination of two or more mAbs increases the antiviral effects by targeting different viral proteins; different mechanisms have been successfully used against Ebola viruses and SARS-CoV-2 (Deeks, 2021; Saxena et al., 2021). Therefore, we addressed the antiviral activity using a combination of 3D8 scFv and NVLH8, which were described as antiviral agents against H1N1/PR8 in two different mechanisms. When neutralizing Abs and 3D8 scFv were used together, the viral genes (HA and NP) were reduced to a greater extent than when they were used separately (Figure 6). While neutralizing candidates inhibited the viral genome releasing steps, 3D8 scFv hydrolyzed viral genomes in the cytoplasm, resulting in additive effects when used together. Further studies are required to develop synergistic effects in *in vivo* models, with considerations of dose and administration methods. Additionally, there is a fact that the neutralizing activity of NVLH8 against H1N1/PR8 was time-dependent, which may come from the low binding affinity to the virus. This feature may lead to low efficiency in *in vivo* experiment. The low binding affinity to critical binding site of the single-domain NVLH8 could be solved by engineering into a bivalent form (Hoang et al., 2022).

In conclusion, our findings show that combining two antibodies with different mechanisms can produce a synergistic effect, potentially providing antiviral activity against influenza viruses. The approach based on a neutralizing antibody that prevents entry of virus into host cells and a catalytic antibody that degrades viral genomes can amplify the inhibition of virus replication cycle.

## Data availability statement

The original contributions presented in the study are included in the article/Supplementary material, further inquiries can be directed to the corresponding authors.

## Ethics statement

Ethical approval was not required for the studies on animals in accordance with the local legislation and institutional requirements because only commercially available established cell lines were used.

## Author contributions

PH: Conceptualization, Investigation, Methodology, Visualization, Writing – original draft, Writing – review & editing. QL: Investigation, Writing – review & editing. RA: Investigation, Writing – review & editing. YL: Investigation, Writing – review & editing. K-JO: Investigation, Writing – review & editing. TK: Supervision, Writing – review & editing. T-KL: Supervision, Writing – review & editing. SL: Conceptualization, Supervision, Writing – review & editing.
