# Supplementary material for: A synergistic therapy against influenza virus A/H1N1/PR8 by a HA1 specific neutralizing single-domain VL and an RNA hydrolyzing scFv
Source: Front Microbiol. 2024 Apr 19;15:1355599. doi: 10.3389/fmicb.2024.1355599 (PMC11066198; doi:10.3389/fmicb.2024.1355599)
Supplement: Supplementary file 1 [file Data_Sheet_1.pdf]

SUPPLEMENTARY MATERIAL

A synergistic therapy against influenza virus A/H1N1/PR8 by a HA1 specific neutralizing single-domain V_L and an RNA hydrolyzing scFv

Phuong Thi Hoang *et al*.

SUPPLEMENTAL TABLES

Table S1

Specific primers for detection of 8 genes segment of influenza H1N1/PR8 used in RT-PCR

**Gene name**	**Forward (5’ – 3’)**	**Reverse (5’ - 3’)**	**Accession No.**
Hemagglutinin (HA)	GGAGCCATTGCCGGTTTTAT	TCCCCATTGATTCCAATTTCAC	NC-002017.1
Neuraminidase (NA)	TGTTCCTGTTACCCTGATACCG	AAGAAATGCTGCTCGCACTAG	NC_002018.1
Nucleoprotein (NP)	ACAGCCAAGTGTACAGCCTA	ATGTCAAAGGAAGGCACGATC	NC_002019.1
Matrix protein (M)	CGCTTTGTCCAAAATGCCC	CCCATTCGTTTCTGATAGGC	NC_002016.1
Polymerase acidic protein (PA)	TGACACCGACGTGGTAAACT	AAAGGTCCCAGGTTCCAGAT	NC_002022.1
Polymerase basic protein 1 (PB1)	AAAGCTGGACTGCTGGTCTC	ATTTTTGCCGTCTGAGCTCTT	NC_002021.1
Polymerase basic protein 2 (PB2)	GAGGTCAGTGAAACACAGGGA	CTTTGCCCAGAATGAGGAATC	NC_002023.1
Nonstructural protein 1 (NS1)	TCTGGACATCGAGACAGCCA	CCGCCATTTCTCGTTTCTGTT	NC_002020.1

Table S2
Strand specific primers used in reverse transcription

**Gene name**	**Sequences (5'-3')**
mRNA-cDNA	TTTTTTTTTTTTTTTT
H1N1/PR8 tag-HA (vRNA) cDNA	**GGCCGTCATGGTGGCGAAT**AGTGCCAAATACGTCAGG
H1N1/PR8 tag-HA (cRNA) cDNA	**GCTAGCTTCAGCTAGGCATC**CAGTCCATCCCCCTTCAATA
H1N1/PR8 tag-NP (vRNA) cDNA	**GGCCGTCATGGTGGCGAAT**CTAGCACGGTCTGCACTCAT
H1N1/PR8 tag-NP (cRNA) cDNA	**GCTAGCTTCAGCTAGGCATC**TCAAAGTCGTACCCACTGGC

Table S3
Strand-specific primers used in qPCR

Gene name	Forward (5’ – 3’)	Reverse (5’ - 3’)
H1N1/PR8 tag-HA vRNA	GGCCGTCATGGTGGCGAAT	CAGTCCATCCCCCTTCAATA
H1N1/PR8 tag-HA cRNA	AGTGCCCAAAATACGTCAGG	GCTAGCTTCAGCTAGGCATC
H1N1/PR8 tag-HA mRNA	AGTGCCCAAAATACGTCAGG	CAGTCCATCCCCCTTCAATA
H1N1/PR8 tag-NP vRNA	GGCCGTCATGGTGGCGAAT	TCAAAGTCGTACCCACTGGC
H1N1/PR8 tag-NP cRNA	CTAGCACGGTCTGCACTCAT	GCTAGCTTCAGCTAGGCATC
H1N1/PR8 tag-NP mRNA	CTAGCACGGTCTGCACTCAT	TCAAAGTCGTACCCACTGGC

Table S4
Primer list for RTqPCR analysis

**Gene name**	**Forward (5' – 3')**	**Reverse (5' - 3')**	**Accession No.**
GAPDH (MDCK Cell Line)	AACATCATCCCTGCTTCCACT	GGCAGGTCAGATCCACAAC	NM_001003142.2
Hemagglutinin (HA)	AGTGCCCAAAATACGTCAGG	CAGTCCATCCCCCTTCAATA	NC_002017.1
Matrix protein1 (M1)	TCCATGGGGCCAAAGAAATC	ACATACCAGGCCAAATGCCA	NC_002016.1
Nucleoprotein (NP)	CTAGCACGGTCTGCACTCAT	TCAAAGTCGTACCCACTGGC	NC_002019.1

SUPPLEMENTAL FIGURES

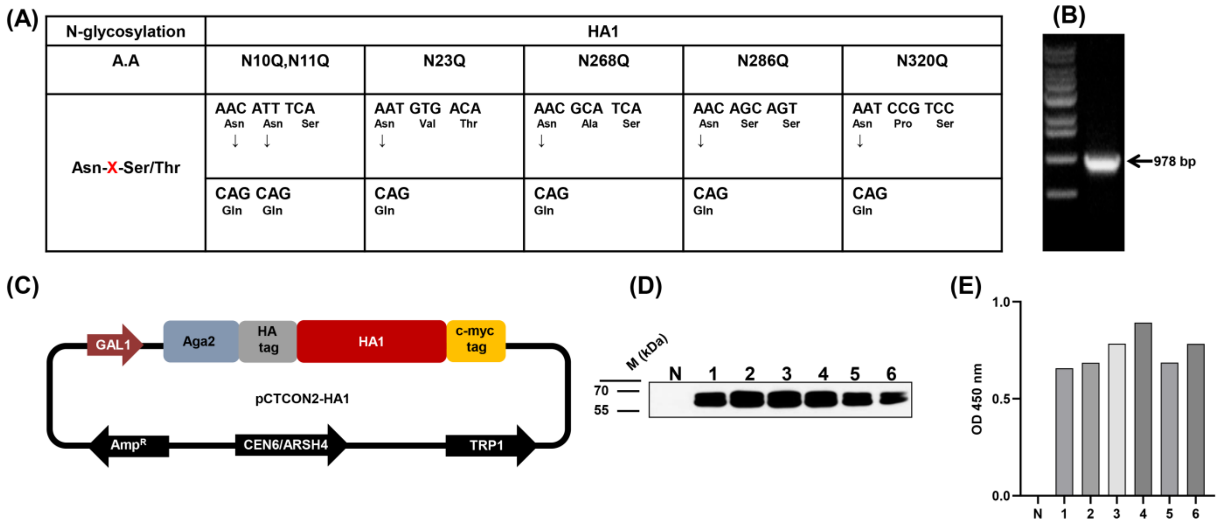

Figure S1. YSD of HA1 antigen of influenza virus A H1N1/PR8

(A) Removal of N-glycosylation sites of HA1 protein with a change at N-glycosylation site, from Asn (N) amino acid to Gln (Q). **(B)** Agarose gel electrophoresis of amplified HA1 DNA by PCR, target product size is 978bp. **(C)** Construction of HA1::YSD in a YSD plasmid, HA1 protein was fused with Aga2 at N-terminal and c-myc tag at C-terminal. Confirmation of HA1::YSD expression by WB **(D)** and ELISA **(E)** with primary anti c-myc antibodies in six different expressing yeast colonies (1–6) and negative control, EBY100 yeast (N).

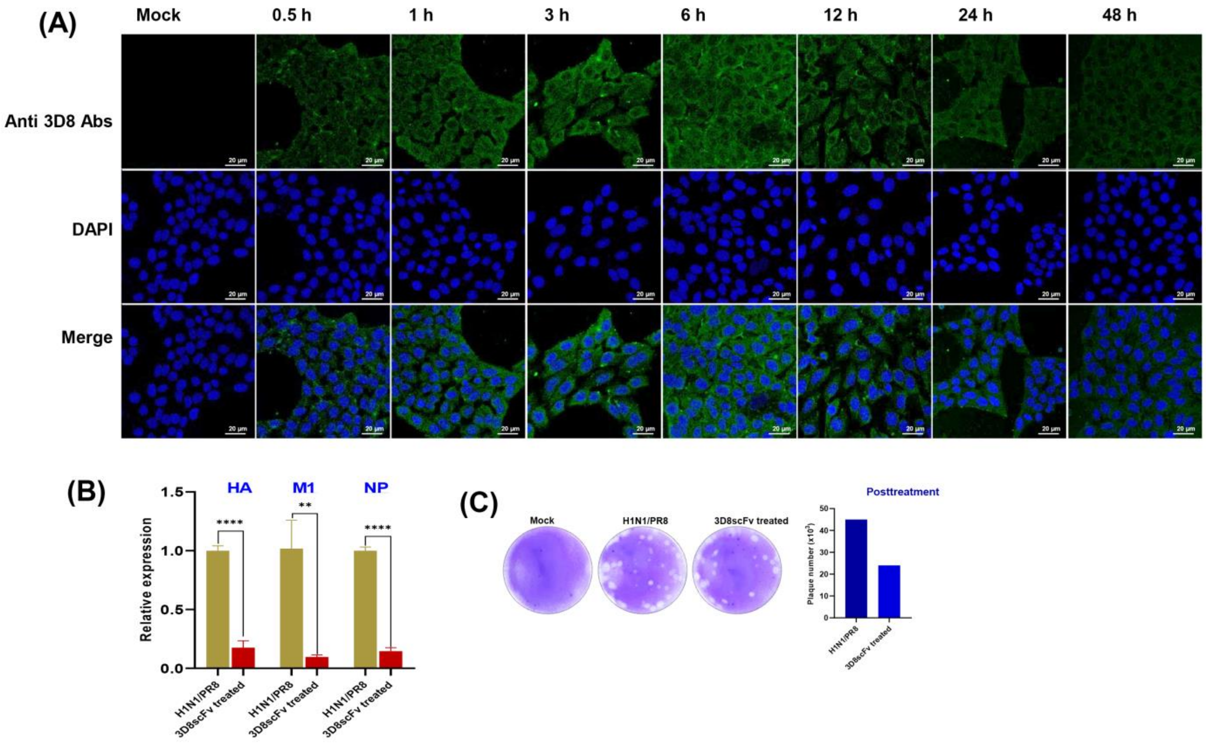

Figure S2. Antiviral activity of 3D8 scFv in a post-viral infection

(A) 3D8 scFv (5 µM) (green) penetrates MDCK cells and remains in cytoplasm till 48 h; nuclei are stained by DAPI (blue). **(B)** HA, M1, NP segment viral gene expression levels were reduced by 3D8 scFv post-treatment compared to those in non-treated groups. Relative gene expression level basing on Delta Delta Ct analysis, graphed by GraphPad prism 8.0, p value by unpaired t test, error bars indicate SD, (*p <0.02, **p<0.005, *** p<0.001 , **** p<0.0001) of triplicate samples. **(C)** Plaque reduction assay of posttreatment, after 24 hpi H1N1/PR8 (MOI 0.1), new progeny virions generated in media supernatant were measured by plaque assay on MDCK cells.

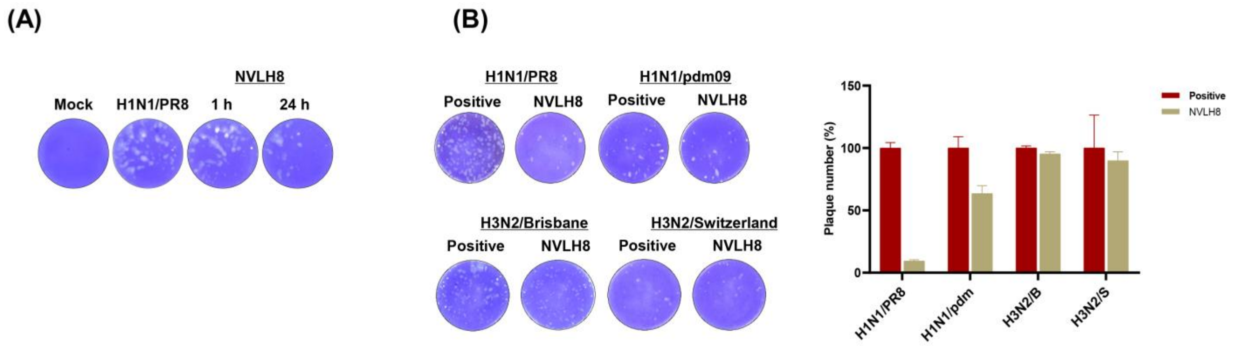

Figure S3. Neutralization activity of NVLH8 specific to H1N1/PR8

(A) NVLH8 showed virus inhibition through plaque assay in 24 h neutralization rather than in 1 h. **(B)** Neutralization of NVLH8 against various strains of influenza virus, H1N1/PR8, H1N1/pdm, H3N2/Brisbane (B) and H3N2/Switzerland (S) in 24 h neutralization assay supporting the specific of NVLH8 against H1N1/PR8. Data was obtained from three independent experiments and graphed by GraphPad prism 8.0, error bars indicate SD.

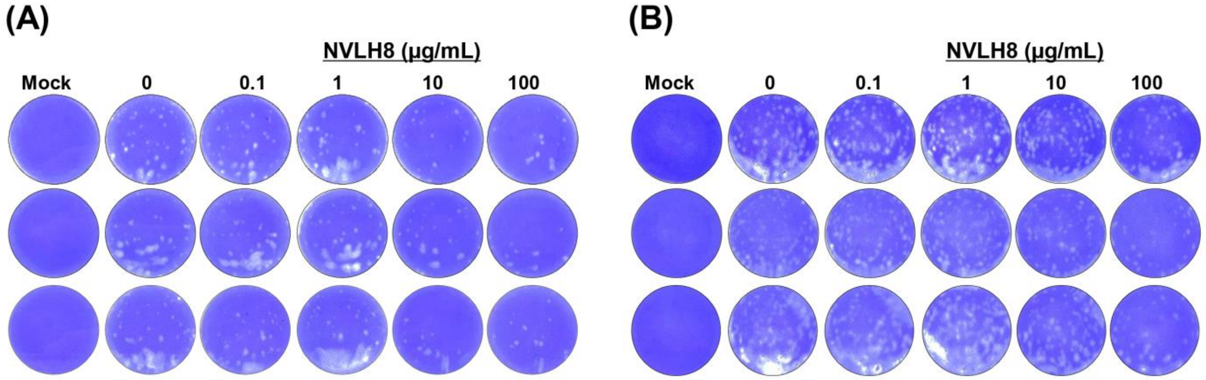

Figure S4. Single-domain V_L (NVLH8) neutralized influenza virus *in vitro* through plaque reduction assay with three independence times for figure 3A. The assay was reproduced either with low titer of viruses (A) or high titer of viruses (B).

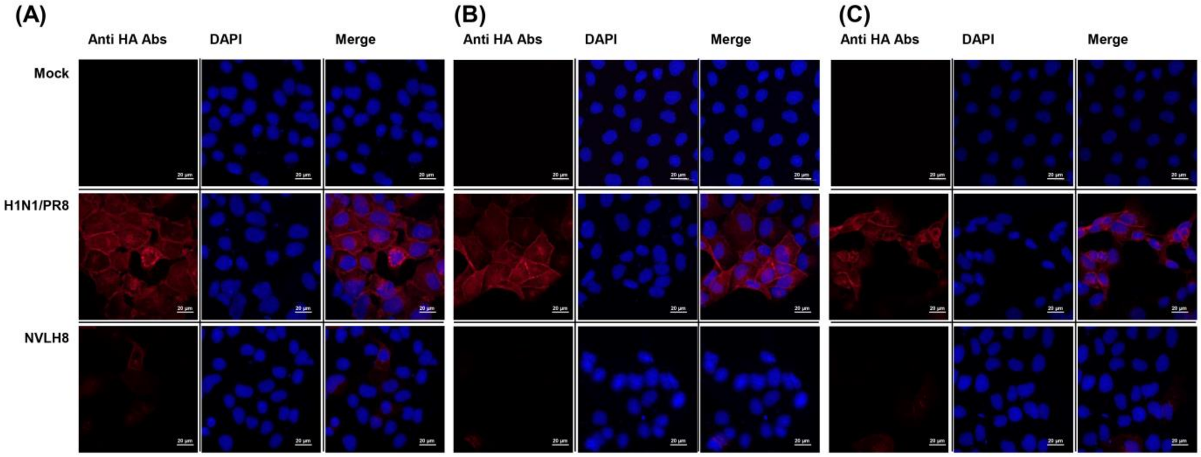

Figure S5. Single-domain V_L (NVLH8) neutralized influenza virus *in vitro* through ICC with three independence pictures (A, B, C) to convert to relative intensity percentages by normalizing to DAPI for Figure 3C.

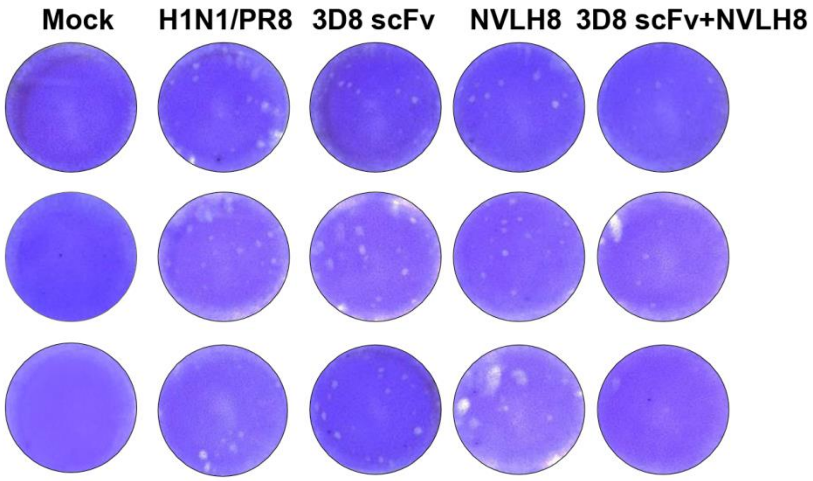

Figure S6. Synergistic effect of neutralizing antibody as a vertical effect and catalytic antibodies 3D8 scFv as a horizontal effect through plaque reduction assay for figure 6C.
